# Recent Trends in the Improvement of the Electrochemical Response of Screen-Printed Electrodes by Their Modification with Shaped Metal Nanoparticles

**DOI:** 10.3390/s21082596

**Published:** 2021-04-07

**Authors:** Karina Torres-Rivero, Antonio Florido, Julio Bastos-Arrieta

**Affiliations:** 1 Departament d’Enginyeria Química, Escola d’Enginyeria de Barcelona Est (EEBE), Universitat Politècnica de Catalunya, BarcelonaTEch (UPC), Av. Eduard Maristany 16, 08019 Barcelona, Spain; karina.torres.rivero@upc.edu (K.T.-R.); antonio.florido@upc.edu (A.F.); 2 Barcelona Research Center for Multiscale Science and Engineering, Av. Eduard Maristany 16, 08019 Barcelona, Spain; 3 Grup de Biotecnologia Molecular i Industrial, Universitat Politècnica de Catalunya, Rambla Sant Nebridi 22, Edifici Gaia TR14, 08222 Terrassa, Spain

**Keywords:** nanoparticles, nanomaterials, shape, screen-printed electrodes, electrochemistry, surface-modification

## Abstract

Novel sensing technologies proposed must fulfill the demands of wastewater treatment plants, the food industry, and environmental control agencies: simple, fast, inexpensive, and reliable methodologies for onsite screening, monitoring, and analysis. These represent alternatives to conventional analytical methods (ICP-MS and LC-MS) that require expensive and non-portable instrumentation. This needs to be controlled by qualified technicians, resulting moreover in a long delay between sampling and high-cost analysis. Electrochemical analysis based on screen-printed electrodes (SPEs) represents an excellent miniaturized and portable alternative due to their disposable character, good reproducibility, and low-cost commercial availability. SPEs application is widely extended, which makes it important to design functionalization strategies to improve their analytical response. In this sense, different types of nanoparticles (NPs) have been used to enhance the electrochemical features of SPEs. NPs size (1–100 nm) provides them with unique optical, mechanical, electrical, and chemical properties that give the modified SPEs increased electrode surface area, increased mass-transport rate, and faster electron transfer. Recent progress in nanoscale material science has led to the creation of reproducible, customizable, and simple synthetic procedures to obtain a wide variety of shaped NPs. This mini-review attempts to present an overview of the enhancement of the electrochemical response of SPEs when NPs with different morphologies are used for their surface modification

## 1. Introduction

Electrochemical methods are advantageous regarding miscellanies instrumental analysis due to their low cost, simplicity, high sensitivity, ease of operation, rapid analysis, portability, and applicability for monitoring different samples in the environmental and pharmaceutical field. In recent years, research in the field of electrochemical sensors has evolved towards the simultaneous analysis of species and miniaturization of electrodes based on new materials and their strategic surface functionalization. Screen printing is a well-established method to produce thick-film electrochemical transducers [1]. This technology is highly reproducible and used for the preparation of single-use screen-printed electrodes (SPEs). SPEs composed of carbon nanoallotropes (e.g., carbon nanotubes, nanofibers, or graphene) represent a versatile sensing tool due to their suitability for incorporation in portable instrumentation [1,2,3]. Additionally, it has been reported how modifying their surface with metal nanoparticles (MNPs) leads to the enhancement of the electrochemical reactivity and sensitivity for specific analytes [4,5,6].

The use of nanoparticles (NPs) to modify Screen Printed Electrodes (SPEs) offers significant advantages in enhancing the mass transference rate and the electrocatalytic activity of the electrode [7,8]. NPs exhibit a higher reactive surface, directly influenced by exposed atoms disposition, which results in more electrocatalytically active sites (edge and corner sites) [9]. This fact provides NPs of different sizes and shapes, preferential reactivity, and selectivity towards electrocatalytically detection of specific analytes, due to different charge distribution or polarization of the shaped entity, during the electrochemical determination [10]. Particularly, some studies have reported using SPEs modified with different MNPs to enhance the sensitivity towards the determination of different toxins in different samples; gold nanoparticles and graphene oxide modified screen printed carbon electrode to detect carbofuran [11], reduced graphene oxide/gold nanoparticles/boronic acid nanocomposite modified screen printed electrode to determine glycoside in food samples [12], Prussian blue nanoparticles-based screen printed electrodes to detect mustard agents [13], a nanocomposite based on gold nanoparticles and graphene oxide quantum to modify screen printed electrodes for the voltammetric determination of Aflatoxin B_1_ [14], and dendritic platinum nanoparticles and gold nanoparticles on screen printed electrode to determine bisphenol A on tap water samples electrochemically [15].

Recently, the importance of well-defined particles and structures (from nanometers to several micrometers) has been recognized in numerous applications, including ceramics, pigments, catalysts, electronics biological labeling, and catalysis [16]. Standard methods for size control employ capping agents such as surfactants, ligands, polymers, or dendrimers to confine the growth in the nanometer scale [17].

Nanotechnology’s development makes it possible to synthesize nanostructures of virtually any shape by chemical strategies or even by 3D printing [18]. This capability to produce nanoparticles with multiple anisotropic (non-spherical) morphologies results in structures with enhanced photoluminescence, different biomolecule interactions, modification of localized surface plasmon resonance, surface charge, and (electro)catalytic performance due to a different electron confinement and the change in electron transport property regarding isotropic (spherical) particles [19,20]. For instance, it is reported that the photocatalytic activity of multiarmed CdS rod particles is higher than spherical particles [19].

Nanoparticles can aggregate during their growth. Ostwald ripening is a growth mechanism where small particles dissolve and are consumed by larger particles [21,22,23,24]. Then, the average nanoparticle size increases with time, and the particle concentration decreases. Therefore, stabilization is required to prevent NPs agglomeration and non-controllable shape or size changes. 

NPs can be obtained by various synthetic routes [23], such as electrochemical methods, decomposition of organometallic precursors, reduction of metal salts in the presence of suitable (monomeric or polymeric) stabilizers, or vapor deposition methods. Sometimes, stabilizers are required to prevent nanoclusters’ agglomeration by providing a steric or electrostatic barrier between particles. In addition, the stabilizers play a crucial role in controlling both the size and shape of nanoparticles [25,26,27].

NP shape control is a complex process requiring a fundamental understanding of the interactions between colloid chemistry, interfacial reactions, and kinetics in which crystal growth must be balanced. There are no accepted mechanisms to explain how shape control works. However, much of the efforts are currently devoted to the controlled growth of metal nanoparticles of different morphologies and the chemical mechanisms behind the generation of particle shapes [28]. Despite this, the morphology control of NPs formation can be achieved by changing experimental parameters, including the concentration of reactants, temperature, pH, and the addition of crystal seeds, stabilizers, oxidation/reduction agents, stirring rate, polymeric supports, and others [29]. Unveiling the different growing and crystallization mechanisms of these nanoparticles is beyond this work.

It is worth saying that most preparation methodologies are based on a seed mediated approach, in which, from a more thermodynamically favorable spherical shape, a preferential growth to more complex nanoparticle geometries is achieved [20,30,31]. The anisotropic crystal formation includes a symmetry-breaking stage, which usually occurs in a complex mixture of salts (precursors, stabilizers, reducing agents) and/or surfactants. The formation of stable nanocrystals relies on the preferential absorption of these molecules (e.g., halides, micelles) on the new facets, as it has been widely reported for anisotropic metal NPs composed particularly by gold, silver, platinum, and palladium [30,31,32,33]. 

**Figure 1:**
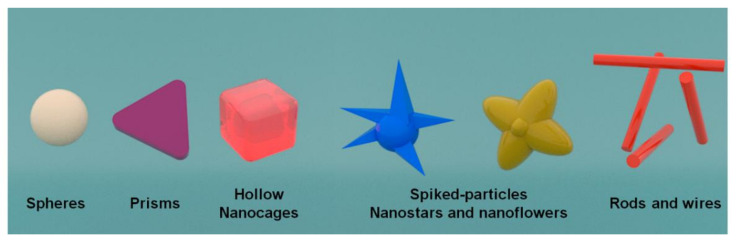
Schematic representation of the different NPs morphologies used for the surface modification of SPEs.

This review focuses on the current trends and advances regarding the modification of SPEs considering different shaped-metal NPs and their electroanalytic applications (see Table 1). Physical and chemical methods are currently used to prepare metal NPs with preferred sizes and morphologies. Physical methods are widely used due to their effectiveness, relative simplicity, and cost effectiveness, combined to the fact that these do not compromise the chemical integrity of the SPE. On the other hand, chemical methods can be more selective and effective by the formation of strong chemical bonds between NPs and SPE, but can in fact, risk the physical stability of the SPE. Therefore, by modifying pH, temperature, reaction time, surfactant concentration, reagent addition rate, capping agents, alternative reduction (galvanic replacement and in situ beam reduction), and precursors, it is possible to obtain metal NPs that exhibit different shapes, such as spherical, rod-like, wire, star, capsules, triangular, tetragonal (Figure 1). As previously commented, NPs’ shape influences their electroanalytical features due to a different atom distribution [6,34,35,36,37,38].

## 2. SPEs Modification by Physical Approaches

Some of the modification techniques rely on the physical incorporation of nanomaterials on SPEs surface without changing their chemical integrity to improve the electrode’s electrocatalytic performance by increasing the effective surface area. Hence, low detection limits, fast responses, high sensitivity, and reproducibility can be obtained with the resulting nano-enabled sensor. 

### 2.1. Drop-Casting Method

Several investigations have been reported using this methodology to modify screen-printed electrodes, using different NPs solutions: copper [51], bismuth [52], gold [53,54], silver.

It is a simple technique where the screen-printed electrode surface is modified by using a solution that can be composed of particles such as nanotubes or nanoparticles [55].

Usually, the NPs solution is placed onto the sensor surface. The solvent evaporation can be performed by introducing the sensor in an oven at a temperature at which the electrode is not damaged. The drop cast amount onto the electrode is the only parameter to be considered for this modification technique [3]. 

Nevertheless, other investigations have reported drawbacks regarding this methodology: the so-called “ring coffee effect” [55,56], where the ring’s periphery concentrates the non-volatile particles in contrast to the center. Marangoni effect, anisotropic nanoparticles, or even surfactants can reduce this effect, creating a uniform distribution of the drop cast nanoparticles on the sensor surface.

### 2.2. Spin Coating

The spin coating method allows producing a uniform distribution of the nanoparticles on the screen-printed electrode surface. This methodology is performed in four steps, (1) deposition in which the material (NPs solution) is deposited onto the sensor’s surface; (2) spin up (acceleration); (3) spin off (deceleration), the applied solution is distributed via centrifugal force, the high spinning speed results in a uniformed layer; and (4) evaporation of the solvent is possible because of rapid rotation [57,58,59]. The coating solution viscosity and the rotation speed controlled the thickness of the deposited layer [57].

### 2.3. Spray Coating

Chomoucka et al. [60] and Mayousse et al. [61] have reported using this method to modify screen printed electrodes. Nanoparticles dispersed in an appropriate solvent (e.g., alcohol) are sprayed onto a substrate through jet/nozzle equipment. The liquid evaporates, allowing the NPs to settle on the surface [62,63].

### 2.4. Sputter Coating

Other investigations have reported the use of this technique to modify the sensor’s surface [64,65]. The sputter coating is referred to the use of the energy of a partially ionized gas (usually argon) on the surface of a target (cathode) to pull out the atoms of the material one by one, and deposit them on the substrate [66].

### 2.5. Electrospray

This method relies on propelling nanoparticles using voltage [62]. The NPs are dispersed into droplets by an existing electrical field between a nozzle and the targeted substrate. The solvent is evaporated before reaching the surface, where the NPs are deposited [67]. Mettakoonpitak et al. [68] reported using electrospray for the deposition of silver nanoparticles on SPEs.

### 2.6. Chemical and Electrochemical Deposition

Chemical deposition takes place when reacting volatile precursors in the gas phase to form a layer that deposits on the desired surface, like SPEs. This approach includes homogeneous reactions occurring in the gas phase and heterogeneous chemical reactions which occur close to a heated surface, forming (nano)powders or (nano)coatings. For example, this strategy was used for the customized preparation of a nanocomposite modified SPE, that consisted of carbon black-Prussian blue NPs [69,70].

Similarly, electrochemical disposition is a modifying technique that can produce nanoparticles with controlled characteristics, size, morphology, and composition on an electrode’s surface [71]. Typically, the oxidized species (metallic salts) are reduced at a fixed potential or current to obtain the metal particles grown on substrates. Using this methodology, it is possible to optimize the precursor solution parameters (e.g., salt type and concentration) and those regarding the electrochemical deposition. Even though bigger particles can be obtained using higher precursor concentrations, two parameters are critical in controlling NPs’ size and shape: the deposition time and the applied potential or current [3].

Many studies have been performed using the electrodeposition and various salts precursor to modify screen-printed electrodes; Ag-NPs and Au-NPs [72] were electrodeposited using AgClO_4_ and HAuCl_4_, respectively. Pt-NPs [73] using K_2_PtCl_6_, Cu-NPs [74] via CuSO_4_, and NiO-NPs [75] employing Ni(NO_3_)_2_·6H_2_O.

### 2.7. Ink Mixing and Printing Method

This technique incorporates into the ink preparation three components: conductive particles usually made of carbonous material, a binder paste mixture, such as resins or cellulose acetate, and even solvents such as terpineol, ethylene glycol, or cyclohexanone [76] that allow the particulate matter to transfer onto the electrode substrate, and the modifying agent, in this particular case, NPs [3]. Sometimes, screen-printed electrodes composed of graphite particles are electrochemically activated to enhance their electrochemical performance [77]. Additionally, a pre-treatment could be performed in carbon-based electrodes to improve the electron transfer rates between the electrode surface and the compounds in solution [76,78].

It is possible to mention some studies reporting the utilization of this modifying technique: nanocomposite consisting of bismuth nanoparticles and amorphous carbon [79], silver and carbon nanoparticles conductive inks [80], silver nanoparticles ink [81,82], and gold nanoparticles ink [83].

## 3. SPEs Modification with Morphologically Different NPs Systems

### 3.1. Spherical Nanoparticles

**Figure 2:**
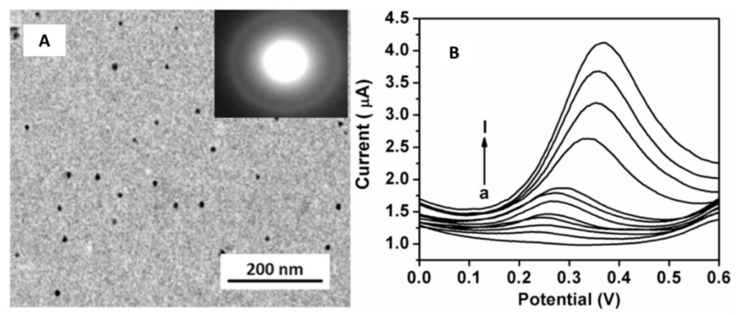
(**A**) TEM micrograph of GR/CD/Pt composite. (**B**) CV response of cysteine at the SPE modified with the GR/CD/Pt in a concentration range of 0.5–170 µM in 0.1 M PBS buffer pH 7.4. Reproduced with permission of Singh et al., Journal of Electroanalytical Chemistry; published by Elsevier, 2018 [39].

Several investigations include the synthesis of nanoparticles with a spherical shape. These NPs have been used to modify SPEs with different applicability in numerous fields. Singh et al. [39] prepared a graphene oxide-cyclodextrin composite with platinum nanoparticles (GR/CD/Pt). This nanocomposite was incorporated into the SPEs by printing it upon the working electrode’s top, obtaining the GR/CD/Pt/SPE, further used for cysteine determination. The modified SPE were characterized by employing scanning electron microscopy (SEM), transmission electron microscopy (TEM), atomic force microscopy (AFM), Fourier transform infrared (FT-IR), and thermogravimetric analysis (TGA). Figure 2A shows a TEM image of the GR/CD/Pt with spherical-shaped particle structure. A coating of platinum NPs over GR/CD composite with an average diameter of 15 nm can be observed.

Moreover, an electrochemical characterization of the GR/CD/Pt/SPE was performed using cyclic voltammetry (CV), differential pulse voltammetry (DPV), and electrochemical impedance spectroscopy (EIS). DPV studies (Figure 2B) exhibited two ranges in which current and cysteine concentrations had a linear correlation from 0.5 to 40 µM and from 40 to 170 µM with a limit of detection (LOD) of 0.12 µM.

**Figure 3:**
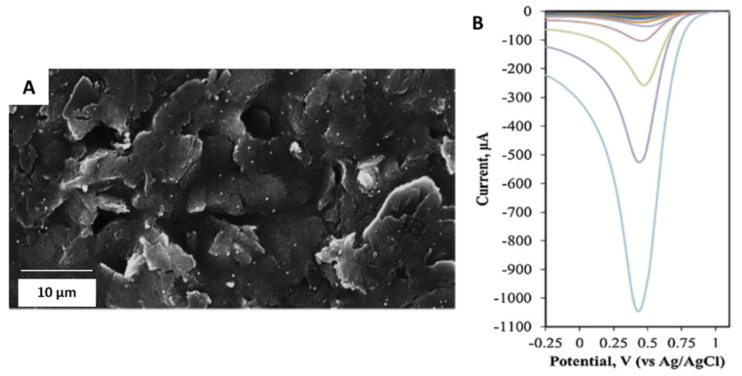
(**A**) SEM micrograph of Rh-NPs deposited on SPEs. (**B**) Cathodic stripping voltammograms of modified electrodes recorded using 150 μL drop of supporting electrolyte with bromide. The cathodic linear sweep voltammetry scan was from 1.11 to −0.25 V at a scan rate of 0.10 Vs^−1^. Reproduced with permission of Cunha-Silva and Arcos-Martínez, Sensors and Actuators B: Chemical; published by Elsevier, 2019 [40].

Other studies carried out by Cunha-Silva and Arcos-Martinez [40] functionalized a SPE with rhodium nanoparticles (Rh-NPs) using the chronoamperometric technique. The obtained sensor was used for bromide anion determination in seawater, surfactant, and pharmaceutical samples. Figure 3A shows that the SPEs surface modified with the electrodeposited rhodium nanoparticles at −0.25 V for 480 s. Figure 3B exhibited the voltammograms obtained using the modified electrodes in 0.05 M phosphate-buffered saline (PBS) with 0.05 M of NaCl as supporting electrolyte. The bromide concentration ranged from 0 to 40 mM.

### 3.2. Triangle Shaped Nanoparticles

Baradoke et al. [41] developed triangular ruthenium nanoplates (Ru-NPLs) to modify graphene screen-printed electrodes to determine *ß*-Nicotinamide adenine nucleotide in its reduced form (NADH), which is related to depression, neurodegenerative diseases (Parkinson and Alzheimer), and even cancer. 

**Figure 4:**
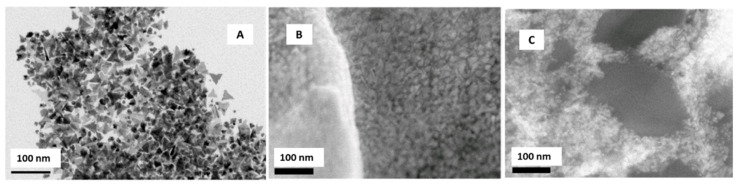
(**A**) TEM micrograph of Ru-NPLs obtained through the hydrothermal reduction of Ru salt by formaldehyde in the presence of PVP. SEM micrographs of Ru-NPLs in H_2_O, (**B**) in ethanol, (**C**) modified screen-printed graphene electrode. Reproduced with permission of Baradoke, Pastoriza-Santos y González-Romero, Electrochimica Acta; published by Elsevier, 2019 [41].

The authors synthesized these Ru nanoplates through a hydrothermal reduction of a ruthenium salt (RuCl_3_·*x*H_2_O) with formaldehyde in the presence of polyvinylpyrrolidone (PVP). TEM micrographs show very thin triangular nanoplates with an edge length of 18 ± 3 nm (Figure 4A).

After the synthesis, the nanoplates were incorporated, using the drop-casting method, to graphene screen-printed electrodes (Ru-NPLs-SPEGPH). Water (Figure 4B) and ethanol (Figure 4C) were used as casting solvents to deposit the Ru-NPLs onto SPEGPH; the first led to the formation of large aggregates of nanoparticles. The second permitted a more homogeneous distribution of the Ru-NPLs. The authors performed a polymerization at pH 7.2 to incorporate the Ru-NPLs on screen-printed carbon electrodes using a poly(o-phenylenediamine) (PoPD) film. Finally, the study of the NADH oxidation on modified SPEGPH was performed. Analytical determination showed that the highest NADH oxidation current was obtained when NADH had direct contact with Ru-NPLs, while the SPEGPH modified with the Ru-NPLs and the PoPD film offered an improved and stable electrocatalytic activity toward the NADH oxidation, exhibiting a very low detection limit (LOD) (4.0 ± 0.9 µM), wide linear range, and good reproducibility.

**Figure 5:**
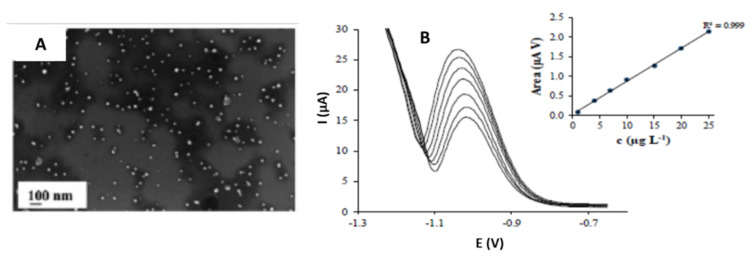
(**A**) SEM micrograph of AgNPr (800 µL of Ag Seeds solution). (**B**) Differential pulse anodic stripping voltammograms of As(V) and its calibration plot in 0.01 mol L^−1^ pH 2 applying −1.30 V and a deposition time of 120 s. Reproduced with permission of Torres-Rivero et al., Nanomaterials; published by MDPI, 2020 [42].

In addition, triangular-shaped nanoparticles were synthesized for the voltammetric determination of heavy metal ions. Torres-Rivero et al. [5,42] synthesized silver nanoparticles (Ag-nanoseeds and Ag-nanoprisms) by the seed mediated approach [84,85]. SEM and TEM images showed that the Ag-nanoprisms had triangular morphology with a size between 14.25 and 16.46 nm (Figure 5A). After the nanoparticle synthesis, the investigators performed a modification onto the screen-printed carbon nanofibers electrodes (SPCNFE) surface by a drop-casting strategy [4,5] using the Ag-nanoseeds (Ag-NS-SPCNFE) and the Ag-nanoprisms (Ag-NPr-SPCNFE). The electrochemical study was completed to verify the enhancement of the voltammetric response provided by silver nanoparticles. In a previous study, the Pb(II) and Cd(II) ions were determined in an acetic acid/acetate buffer [5]. In contrast, in another investigation, As(V) ions were detected in a HCl electrolyte [42] using the differential pulse anodic stripping voltammetry technique.

To perform the electrochemical study, As(V) ions were deposited at a deposition potential of −1.3 V for a deposition time of 120 s. The scanning potential was from −1.2 to −0.6 V, where the As(V) peak was exhibited at −1.0 V (Figure 5B).

The authors observed an excellent linear response between the peak area and the As(V) concentration. The researchers also pointed out that even the obtained limits of detection using Ag-NPr-SPCNFE (1.2 and 2.6 µg·L^−1^) were lower than similar studies. The linear range’s highest limit is restricted to a lower concentration value (25 µg·L^−1^). Finally, the modified electrode was tested in spiked water samples obtaining results comparable to those obtained with inductively coupled plasma-mass spectrometry (ICP-MS) measurements.

### 3.3. Star-Shaped Nanoparticles

**Figure 6:**
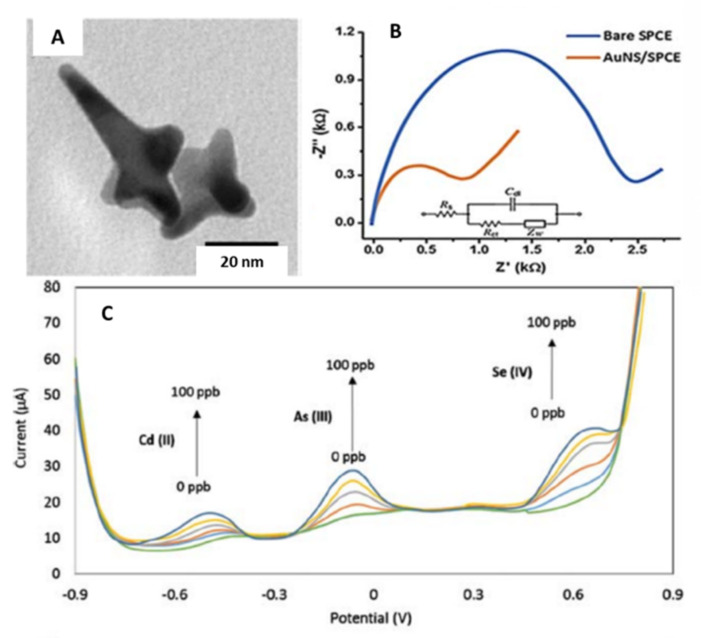
(**A**) TEM micrograph of gold nanostars. (**B**) Nyquist plot of the bare and gold nanostar modified screen-printed electrode in the presence of the redox probe 10 mM Fe(CN)_6_^3−/4−^. (**C**) SWASV curves of the AuNS-modified SPCE for the simultaneous detection of Cd(II), As(III), Se(IV) in a concentration range of 0 to 100 μg L^−1^. Reproduced with permission of Lu et al., Analytical and Bioanalytical Chemistry; published by Springer, 2020 [43].

In addition to the traditional shapes, new and novel shaped nanoparticles have been developed. Lu et al. [43] synthesized gold nanostar (Au-NS) to modify screen-printed carbon electrodes (SPCE) for the simultaneous detection of Cd(II), As(III), and Se(IV). The morphology and size of the Au-NS were estimated using TEM images (Figure 6A). The average tip-to-tip diameter was 49 ± 14 nm, and the number of spikes per nanostar ranged from 4 to 10. Additionally, the behavior of the gold nanostars on the SPCE was studied using electrochemical impedance spectroscopy. The charge transfer resistance decreased significantly from 2.4 kΩ (bare electrode) to 0.8 kΩ (Au-NS-SPCE) (Figure 6B). This difference was related to the augmented area due to the Au-NS coating the SPE surface.

The modified electrode was used to perform an electrochemical study using the Britton–Robinson buffer (BRB). The boric acid was excluded from the buffer, resulting in a modified solution of equal amounts of phosphoric and acetic acid (0.1 M pH 2.0) (mBRB). Square wave anodic stripping voltammetry (SWASV) was used to detect Cd(II), As(III), and Se(IV) simultaneously. They were deposited using a deposition potential of −0.9 V for a deposition time of 180 s. The stripping potential was from −0.9 to 0.9 V, with an amplitude of 70 mV, a period of 20 ms, a step increment of 11 mV, and a sampling width of 5 ms. Figure 6C shows the corresponding voltammograms of the simultaneous detection of the mentioned metal ions. Cd(II), As(III), and Se(IV) exhibited peaks at approximately −0.48, −0.09, and 0.65 V (vs. Ag/AgCl), respectively. The obtained LODs were 1.62, 0.83, 1.57 µg·L^−1^ for Cd(II), As(III), and Se(IV), respectively. However, the authors reported the formation of arsenic triselenide (As_2_Se_3_), which is a highly stable and insoluble compound that could affect the stripping response of the As(III) and Se(IV). Finally, the Au-NS-SPCE was tested with real water samples. The results showed the proposed method could represent a reliable method to detect Cd(II), As(III), and Se(IV) simultaneously in environmental samples. 

Dutta et al. [44] presented the gold nanostars synthesis by Good’s buffer method [86,87], which was used to modify a carbon paste screen-printed electrode (CPSPE) for the electrochemical detection of Cr(VI) in water.

**Figure 7:**
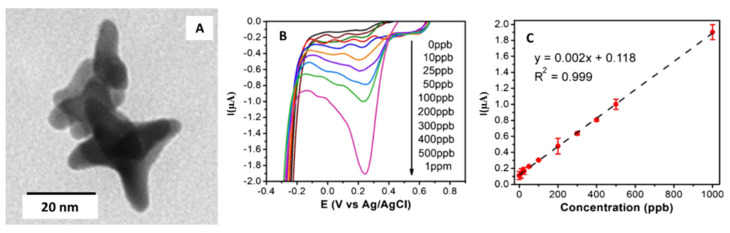
(**A**) TEM micrograph of gold nanostars. (**B**) Linear sweep voltammograms obtained with the SPE modified with the gold nanostar in 0.1 M H_2_SO_4_ with Cr(VI) additions between 0 and 1000 μg·L^−1^. (**C**) Calibration curve obtained for the Cr(VI) additions. Reproduced with permission of Dutta et al., Microchimica Acta; published by Springer, 2019 [44].

Figure 7A shows a TEM micrograph of the synthesized Au-NS. The diameter of the Au-NS inner sphere, which ranged from 10 to 22 nm. Additionally, the star diameter ranged from 30 to 52 nm.

CPSPEs were modified by drop-casting with increasing quantities of Au-NS solutions (from 7.5 to 66 µL). The authors determined that the optimal amount of Au-NS solution was 22 µL, which offered the highest current density for Cr(VI). The modified electrode was used to detect Cr(VI) in water using linear sweep voltammetry (LSV) (see Figure 7B); also, a linear relationship between the current and the Cr(VI) concentration is observed (see Figure 7C). The potential was scanned from −0.7 to 0.8 V with a scan rate of 0.05 V·s^−1^. All measurements were performed in 0.1 M sulfuric acid. The limit of detection and quantification were 3.5 and 10 µg·L^−1^, respectively. Electrode sensitivity was found to be 20 nA ppb^−1^cm^−2^.

In addition, a study with the presence of possible interferents, Ni(II), Zn(II), Fe(III), Cr(III), Pb(II), As(III), Cu(II), Se(IV), and Cd(II) was performed. The authors studied the response of the modified CPSPE with 100 µg·L^−1^ Cr(VI) and 1 mg L^−1^ of each metal ion. They could observe no significant change in the LSV peak current value in the presence of metal ions.

Finally, a determination of Cr(VI) in contaminated groundwater was carried out. The results were contrasted with ICP analyses to assess the accuracy of the voltammetric sensor. Recoveries percentages ranged from 95% to 97%.

### 3.4. Nanoflowers Shaped Nanoparticles

Glycated hemoglobin (HbA1c) is now considered a promising biomarker for the diagnosis of type II diabetes (T2D) [88,89]. Wang et al. [45] developed an electrochemical biosensor using a screen-printed electrode modified with gold nano-flowers (AuNFs) to quantify the HbA1c.

**Figure 8:**
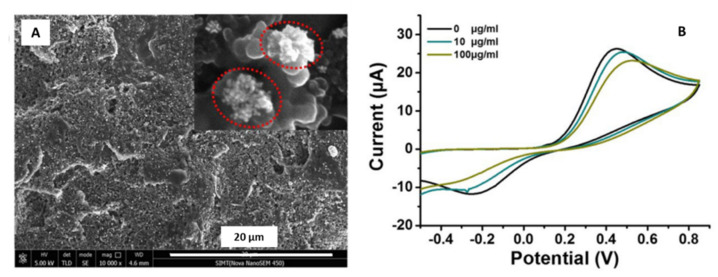
(**A**) SEM micrograph of gold nanoflowers deposited onto the Screen Printed Carbon Electrode (SPCE). (**B**) Cyclic voltammograms obtained for the modified electrode detecting HbA1c in 2.5 mM K_4_Fe(CN)_6_ containing 0.1 M KCl. Reproduced with permission Wang et al., Talanta; published by Elsevier, 2019 [45].

AuNFs were electrochemically deposited on the screen-printed carbon electrode (Figure 8A). A capture molecule (4-Mercaptophenylboronic acid or 4-MPBA) was used to catch the HbA1c; mediated by the boric acid and the 4-MPBA, interacting with the target sugar subunit HbA1c. Once the HbA1c was immobilized on the SPCE, it could produce a reduction of H_2_O_2_ due to its catalytic property. This allows the study of the electrochemical response, as there is a proportionality between the amount of the captured HbA1c and the reduced H_2_O_2_ on the modified electrode (see Figure 8B).

The voltammetric results confirmed the modified electrode’s successful application to quantify the glycated hemoglobin in the range between 5 and 100 µg·mL^−1^. The proposed electrode was also tested in human blood, reaching a recovery rate between 99% and 103.8%. The authors suggested a promising potential method to monitor real samples of diabetes patients and are extended to detect glycoprotein biomarker of other chronic diseases, such as cancer.

Other studies used rare earth elements combined with metal oxide nanocomposites to develop novel nanostructures, enhancing the catalytic activity to fabricate efficient sensors. In that sense, Rezaei et al. [46] synthesized lanthanum-doped zinc oxide nanoflowers to modify a graphite screen-printed electrode for the detection of hydrochlorothiazide (HCT). The HCT is a drug extensively used for hypertension treatment, increasing the excretion of sodium chloride and water from the kidney [90]. The HCT is also used for heart failure treatment, liver cirrhosis, and kidney disorders [91]. The authors prepared the La^3+^-doped ZnO nanoflowers using nano-powders: zinc acetate, lanthanum nitrate, and thiourea ammonia. This last reagent was used as a complexing agent.

**Figure 9:**
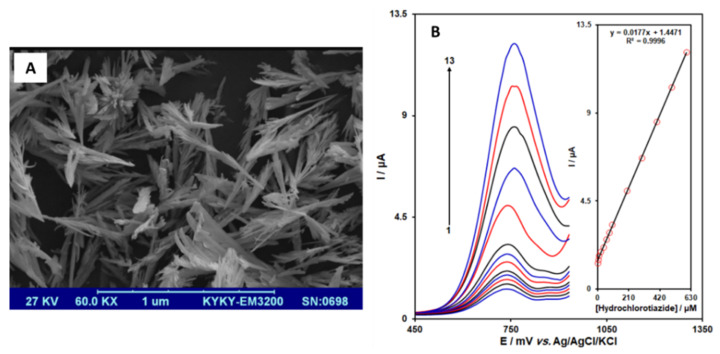
(**A**) SEM micrograph of La^3+^-doped ZnO nanoflowers. (**B**) Differential pulse voltammograms obtained for the La^3+^/ZnO/SPE in the presence of different concentrations ranging from 1 to 600 µM of hydrochlorothiazide. Inset figure corresponds to the calibration plot for the HCT determination. Reproduced with permission of Rezaei et al., International Journal of Electrochemical Science; published by ESG, 2019 [46].

After the nanoflowers synthesis, they were characterized by SEM, as shown in Figure 9A.

Graphite screen-printed electrodes were modified by the drop-casting strategy. The modified sensor was characterized using cyclic voltammetry and differential pulse voltammetry (DPV) (See Figure 9B). Firstly, a pH study was performed. The authors concluded that HCT is a pH-dependent molecule, determining the higher oxidation current values for hydrochlorothiazide occurred at pH 7.0.

DPV measurements were performed in 0.1 M phosphate buffer saline (PBS) containing different concentrations of HCT, in a range from 1.0 to 600.0 µM. The limit of detection was 0.6 µM. Finally, the La^3+^/ZnO/SPE was used to evaluate the proposed method’s applicability to determine HCT in tablets and urine samples. The results showed that recoveries ranged from 98% to 103%, with excellent reproducibility.

### 3.5. Nanowires

Usually, SPE are modified with nanowires for different purposes: biomedical, environmental, and food industry. In particular, nanowires are capable of interfacing with other nano-micro scale systems. Due to the long axial morphology, nanowires have a higher surface-to-volume ratio making them similar to biological macromolecules to create excellent nano-bio devices [92]. Kabir et al. [47] developed an electrochemical sensor to detect phosphate using novel ammonium molybdate tetrahydrate/silver nanowires (AMT/AgNWs) modified SPE. 

The authors prepared the AgNWs following the procedure developed by Korte et al. [93]. AgNWs were synthesized using silver nitrate as a precursor and polyol as a reducing agent. Additionally, CuCl or CuCl_2_ were added to reduce the remaining free Ag^+^ ions during the initial phase of AgNWs formation. 

**Figure 10:**
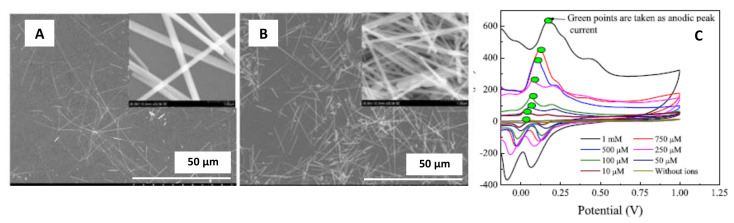
(**A**) SEM micrograph of ammonium molybdate tetrahydrate (AMT)/silver nanowires (AgNWs) for a reaction time of 10 min. (**B**) (AMT)/silver nanowires (AgNWs) for a reaction time of 16 min. (**C**) Cyclic voltammograms obtained for the AMT/AgNWs modified screen-printed electrodes for phosphate detection using 0.1 M H_2_SO_4_/KCl electrolyte. Reproduced with permission of Kabir et al., IEEE Sensors Journal; published by IEEE, 2018 [47].

After the synthesis, the investigators modified a screen-printed electrode with the AgNWs and AMT using the drop-casting method [5]. The modified electrode surface was characterized using SEM; the AgNWs exhibited a 100 nm diameter approximately for a reaction time of 10 min. In comparison, a reaction time of 16 min generated AgNWs with a larger diameter of 125 nm (Figure 10A,B).

The AMT/AgNWs/SPE were electrochemically characterized using cyclic voltammetry, with a sweep potential from −0.4 to +0.4 V and a scan rate of 50 mV·s^−1^ (see Figure 10C). The results allowed the authors to conclude that AgNWs contributed in increasing the anodic peak current. The calibration curves exhibited linearity between the anodic peak current and the phosphate concentration. Therefore, the use of AgNWs increased the sensitivity of the modified SPE, reaching a sensitivity of 0.71 µA·µM^−1^. Additionally, the LOD value was found to be 3 µM.

### 3.6. Nanocages

Nobel-metal nanocages represent a novel type of nanostructures with hollow interiors and porous walls [94]. These structures are produced by galvanic replacement reaction, resulting in assemblies with unique and tunable properties. Compared to the solid nanoparticles, both inner and outer surfaces of gold nanocages (AuNCs) provide good electron transfer from the aptamers’s (short DNA or RNA fragments) redox center to the surface electrode [48].

**Figure 11:**
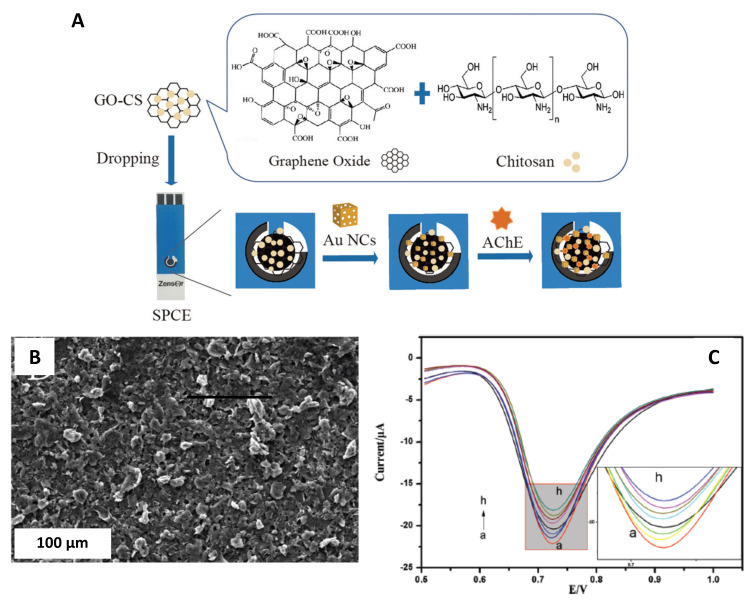
(**A**) Schematic process to construct AChE sensor. (**B**) SEM micrograph of the AuNCs/GO-CS nanocomposite. (**C**) DPV obtained for AChE/AuNCs/GO-CS/SPCE in PBS buffer pH 8.0 with 1.0 mM ATCl after inhibition with chlorpyrifos (0.01, 0.1, 1, 5, 10, 50, 100, and 500 µg·L^−1^) for 12 min. Reproduced with permission of Yao et al., New Journal of Chemistry; published by The Royal Society of Chemistry, 2019 [48].

Yao et al. [48] developed a new biosensor to detect chlorpyrifos, an extensively used organophosphate pesticide in agriculture [95]. Firstly, a nanocomposite was constructed of graphene oxide (GO), chitosan (CS), and the AuNCs. Secondly, the acetylcholinesterase (AChE) enzyme was immobilized in the previous matrix and was used to modify a screen-printed electrode. Finally, the constructed biosensor AuNCs/GO-CS/AChE/SPCE (Figure 11A) had good sensitivity towards detecting acetylthiocholine chloride (ATCl) and pesticides.

Several characterization techniques such as SEM, TEM, high-resolution scanning transmission electron microscopy (HR-STEM), X-ray diffraction (XRD), energy dispersive spectroscopy (EDS), among others, were used to characterize the nanocomposite and the AuNCs (see Figure 11B).

The electrochemical response of the AuNCs/GO-CS/AChE/SPCE was studied by cyclic voltammetry in phosphate buffer containing 0.1 M KCl and 5 mM [Fe(CN)_6_]^3−/4−^ (see Figure 11C). The cyclic voltammograms exhibited that the peak current signal increased with the AuNCs, promoting the electron transfer. Additionally, electrochemical impedance spectroscopy (EIS) studies were performed on the modified biosensor. These results exhibited that after immobilization of the AChE, the impedance was significantly reduced compared to bare SPCE.

### 3.7. Nanocubes

Among the variety of shaped nanoparticles, cubic nanoparticles have received particular interest because of their intrinsic size-dependent properties and resulting applications [96], i.e., silver nanocubes have been used for several applications, including plasmonic sensing surface-enhanced Raman scattering, metamaterials, catalysis, and bionanotechnology [97].

Sudan I (1-phenylazo-2-naphthol) is an industrial dye used to color oils, waxes, and polishes, but also it is added to food and cosmetics for color enhancement [98]. This dye can have a genotoxic effect and also can be a potential carcinogen. Food adulteration with this substance is considered a significant risk for public health [99].

**Figure 12:**
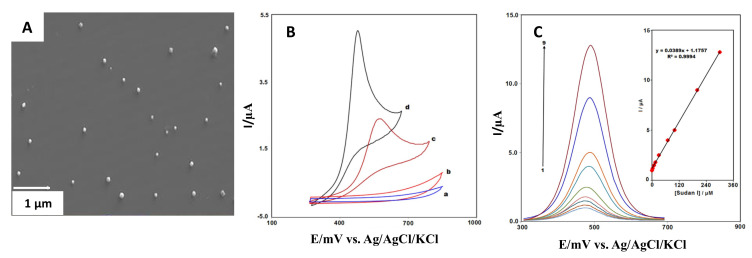
(**A**) La^3+^-doped Co_3_O_4_ nanocubes SEM micrograph. (**B**) Cyclic voltammograms obtained for the bare (a, c) and modified (b, d) SPE in presence and absence of Sudan I dye. (**C**) Differential pulse voltammograms of La^3+^-doped Co_3_O_4_ nanocubes/SPE for different Sudan I concentrations (0.3–300 µM). Reproduced with permission of Mahmoudi-Moghaddam et al., Food Chemistry Journal; published by Elsevier, 2019 [49].

Mahmoudi-Moghaddam et al. [49] developed a screen-printed electrochemical sensor based on La^3+^-doped Co_3_O_4_ nanocubes to determine the Sudan I dye. The La^3+^-doped Co_3_O_4_ nanocubes (Figure 12A) were synthesized using cobalt(II) nitrate hexahydrate Co(NO_3_)_2_·6H_2_O, lanthanum(III) nitrate hexahydrate La(NO_3_)_3_·6H_2_O, and polyvinylpyrrolidone (PVP). After the synthesis, the screen-printed electrodes were modified following a drop-casting method.

Previous sample preparation was performed to study Sudan I electrochemical response. First, a cyclic voltammetry study was completed. As Figure 12B shows, the analyses conducted with the La^3+^-doped Co_3_O_4_ nanocubes/SPE significantly increased the electrode’s electrochemical activity for analyzing Sudan I.

Figure 12C exhibited the differential pulse response corresponding for La^3+^-doped Co_3_O_4_ nanocubes/SPE. The calibration curve shows a linear correlation between the modified electrode’s peak current and the different Sudan I concentrations. These results showed an excellent analytical performance with a LOD and LOQ of 0.05 and 0.15 µM, respectively.

Another investigation used iron oxide nanocubes (Fe_2_O_3_-NCs) to modify screen printed electrodes (Fe_2_O_3_-NCs-SPE) and determine Meclizine electrochemically [50]. Meclizine is an antihistamine drug commonly used to help with motion sickness and dizziness [100]. The authors synthesized iron oxide nanocubes using a hydrothermal approach with ferric chloride (FeCl_3_·6H_2_O as a precursor. Once the nanocubes were obtained, the drop-casting technique was used to modify the screen-printed electrodes with SDS molecules’ addition. SDS is an anionic surfactant that forms a monolayer on the SPE surface with a high density of negatively charged ends. This effect can probably enhance the voltammetric signal of MEC in highly acidic media [50].

**Figure 13:**
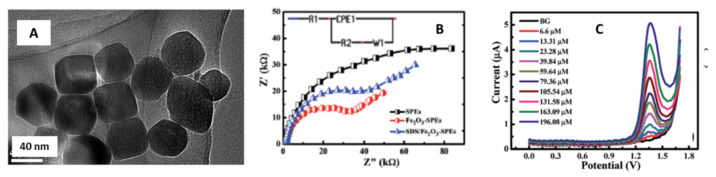
(**A**) Fe_2_O_3_ nanocubes HR-TEM micrograph. (**B**) Nyquist plots obtained for the non-modified SPEs, Fe_2_O_3_-NCs-SPEs and SDS/Fe_2_O_3_-NCs-SPEs. (**C**) Differential pulse voltammograms of Fe_2_O_3_ nanocubes/SPE for different meclizine concentrations (6.6–196.08 µM). Reproduced with permission of Khorshed et al., Analytical Methods Journal; published by The Royal Society of Chemistry, 2019 [50].

In Figure 13A, a high-resolution transmission electron microscopy (HR-TEM) micrograph of the synthesized Fe_2_O_3_-NCs is observed, an average particle size of 37 nm was obtained.

The modified SPE’s electrochemical behavior was studied using several electrochemical techniques, impedance spectroscopy, cyclic voltammetry, and differential pulse voltammetry. 

The Nyquist plots (Figure 13B) exhibited a successful attachment of the Fe_2_O_3_-NCs onto the SPE surface, decreasing the charge transfer resistance significantly compared to the non-modified electrode.

The differential pulse voltammograms (Figure 13C) were obtained in 0.05 M H_2_SO_4_ and increasing MEC concentration ranging from 6.66–196.08 µM. The calibration plot confirmed the linearity between the oxidation peak heights and the MEC concentration, with a limit of detection of 1.69 µM. 

Finally, the modified SPEs were used to analyze real samples (pharmaceutical formulation and urine), showing recoveries of 99.28% and over 100%, respectively. This reveals the potential applicability of Fe_2_O_3_-NCs-SPE for the meclizine determination.

## 4. Conclusions and Future Perspectives

The progress in synthetic approaches has led to the preparation of a wide variety of shaped nanoparticles. Nevertheless, we consider that after summarizing different examples in this work, there is a lack of systematic comparison of the different morphologies of the same metal (like gold or silver) regarding their electrocatalytic response towards the same or different analytes, which could open a brand-new kind of simultaneous electroanalysis platforms based on these nanomaterials.

The presented examples of SPEs surface modification with shaped NPs prove the enhancement effect of their electrochemical response. One step beyond this trend would be to tune these shaped particles’ physical and chemical properties with a Janus particle configuration. This means NPs with asymmetry in terms of physical or chemical properties that would make possible the preparation of simultaneous and more specific sensing systems; aimed by preparing Janus particles with customized-differential features: chemical composition, hydrophobicity, roughness, hardness, and surface charge. These multifunctional electrocatalytic materials could be incorporated into bioinspired sensor systems (like electronic noses and tongues), determining and quantifying simultaneously different analytes. 

Chemical contamination of surface waters and surrounding soil, continuously increases as the human way of life improves and as hydric resources decrease as a consequence of global warming and climate change. This is a serious threat not only for humans, but also for aquatic organisms and ecosystems. Innovative methodologies are necessary for the screening and monitoring of the considered substances in natural waters, wastewaters, and food products with lower cost, simpler and faster operation analytical techniques, ready for in situ analysis, and able to inform about chemical speciation, which in some elements is closely related to toxicity. In that sense, the NP modified SPEs represent a versatile sensing tool for their feasible incorporation in portable instrumentation due to their enhanced electroanalytical performance towards analytes that can be found in water source and are considered of environmental interests like heavy metals or pharmaceutical residues.

It would then be interesting to evaluate their suitability for the simultaneous analysis of chemical species usually found in natural waters, wastewaters, and food–daily used products. One clear example is pharmaceutical residues, mainly due to the rapid increase in pharmaceutical products’ consumption by the human population and farm animals.

## Figures and Tables

**Table 1:** Summary of relevant shaped NPs and their electroanalytical application.

Metal/Shape.	Size	SPE/Modification Strategy	Analyte	LOD	Real Samples Study	Reference
Pt/Sphere	15 nm	Reduced graphene screen-printed electrode/Ink mixing	Cysteine	0.12 µM	NA	Singh et al. [39]
Rh/Sphere	Not specified	Screen printed carbon electrode (SPCE)/Electrodeposition	Bromide anion	39 µM	Seawater, surfactant, pharmaceutical formulation	Cunha-Silva and Arcos-Martinez [40]
Ru/Triangles	Approx. 18 nm	Graphene modified screen printed carbon electrode (SPEGPH)/Drop-casting	Biomedicine/ß-Nicotinamide adenine nucleotide (NADH)	4.0 µM	NA	Baradoke et al. [41]
Ag/Triangles	Between 14.25 and 16.46 nm	Screen printed carbon nanofibers electrode (SPCNFE)/Spincoating	Voltammetric determination of As (V)	1.6–2.5 µg·L^−1^	Tap water	Torres-Rivero et al. [42]
Gold/Star	Tip-to-tip diameter 49 ± 14 nm, spikes number nanostar ranged from 4 to 10 nm.	Screen printed carbon electrodes (SPCE)/Drop-casting	Simultaneous detection of Cd (II), As (III), and Se (IV)	1.62, 0.83, 1.57 µg·L^−1^ for Cd(II), As(III) and Se(IV), respectively	Ground and Surface water	Lu et al. [43]
Gold/Star	Star diameter ranged from 30 to 52 nm	Carbon pasted screen printed electrode (CPSPE)/Drop-casting	Detection of Cr (VI) in water	3.5 µg·L^−1^	Groundwater	Dutta et al. [44]
Gold/Nanoflowers	Not specified	Screen printed carbon electrode (SPCE)/Electrodeposition-drop-casting	Glycated hemoglobin (HbA1c)	0.65%	Human blood	Wang et al. [45]
Lanthanum-doped zinc oxide/Nanoflowers	Not specified	Graphite screen printed electrode/Drop-casting	Hydrochlorothiazide	0.6 µM	Pharmaceutical formulation and urine	Rezaei et al. [46]
Ammonium molybdate tetrahydrate silver/Nanowires	Diameter of 100 nm for a reaction time of 10 min	Carbon screen printed electrode/Drop-casting	Phosphate detection	3 μM	NA	Kabir et al. [47]
GO-CS/AChE/Gold/Nano-cages	20–50 nm, lattice spacing distances along the adjacent fringes were 0.235 nm	Screen printed carbon electrode (SPCE)/Drop-casting	Chlorpyrifos detection	3 ng·L^−1^	Vegetable samples	Yao et al. [48]
Lanthanum-doped Co_3_O_4_/nanocubes	Not specified	Graphite screen printed electrode/Drop-casting	Sudan I	0.05 µM	Food samples	Mahmoudi-Moghaddam et al. [49]
Fe_2_O_3_/nanocubes	37 nm	Carbon-graphite screen printed electrode/Drop-casting	Meclizine	1.69 µM	Pharmaceutical formulation	Khorshed et al. [50].

## Data Availability

Not applicable.
